# Subarachnoid hemorrhage: tests of association with apolipoprotein E and elastin genes

**DOI:** 10.1186/1471-2350-8-49

**Published:** 2007-07-31

**Authors:** Ritesh Kaushal, Daniel Woo, Prodipto Pal, Mary Haverbusch, Huifeng Xi, Charles Moomaw, Padmini Sekar, Brett Kissela, Dawn Kleindorfer, Matthew Flaherty, Laura Sauerbeck, Ranajit Chakraborty, Joseph Broderick, Ranjan Deka

**Affiliations:** 1 Department of Environmental Health, Center for Genome Information, University of Cincinnati, Cincinnati, Ohio, USA; 2 Department of Neurology; University of Cincinnati College of Medicine, Cincinnati, Ohio, USA

## Abstract

**Background:**

Apolipoprotein E (*APOE*) and elastin (*ELN*) are plausible candidate genes involved in the pathogenesis of stroke. We tested for association of variants in *APOE *and *ELN *with subarachnoid hemorrhage (SAH) in a population-based study. We genotyped 12 single nucleotide polymorphisms (SNPs) on *APOE *and 10 SNPs on *ELN *in a sample of 309 Caucasian individuals, of whom 107 are SAH cases and 202 are age-, race-, and gender-matched controls from the Greater Cincinnati/Northern Kentucky region. Associations were tested at genotype, allele, and haplotype levels. A genomic control analysis was performed to check for spurious associations resulting from population substructure.

**Results:**

At the *APOE *locus, no individual SNP was associated with SAH after correction for multiple comparisons. Haplotype analysis revealed significant association of the major haplotype (Hap1) in *APOE *with SAH (*p *= 0.001). The association stemmed from both the 5' promoter and the 3' region of the *APOE *gene. *APOE *ε2 and ε 4 were not significantly associated with SAH. No association was observed for *ELN *at genotype, allele, or haplotype level and our study failed to confirm previous reports of *ELN *association with aneurysmal SAH.

**Conclusion:**

This study suggests a role of the *APOE *gene in the etiology of aneurysmal SAH.

## Background

Non-traumatic, spontaneous subarachnoid hemorrhage (SAH) affects 16,000 to 17,000 individuals each year in the United States [1-3]. SAH has a 30-day mortality rate exceeding 40%, and surviving patients often demonstrate significant morbidity [2,4]. Over 80% of SAH can be attributed to intracranial aneurysm (IA) rupture. Familial aggregation studies of SAH have consistently identified an increased risk of a first-degree relative with SAH or family history of SAH independent of smoking and hypertension [5].

Variants of the apolipoprotein E (*APOE*) gene have been associated with Alzheimer's disease, lipid disorders and cardiovascular disease [6-8]. Previous studies have demonstrated that *APOE *ε4 and/or *APOE *ε2 are associated with lobar intracerebral hemorrhage (ICH) [9,10]. We recently reported that haplotypes which include polymorphisms in the 5' untranslated region of the *APOE *gene are risk factors for lobar ICH [11]. Specific to SAH, Kokubo *et al*. [12] found significant association of *APOE *ε4 with SAH in a Japanese population. Niskakangas *et al*. [13] reported association of *APOE *ε4 with adverse outcome after aneurysmal SAH. No study on other polymorphisms of *APOE *with regard to risk of SAH has yet been reported.

In addition to *APOE*, the elastin (*ELN*) gene emerged as a putative gene for IA after linkage was found on 7q11, where *ELN *is located [14]. However, prior association studies of SNPs in *ELN *have been contradictory and remain inconclusive [15,16]. Further, few studies have been performed in US populations.

We performed a case-control study examining the association of variants in *APOE *and *ELN *among a group of US Caucasians with SAH.

## Methods

### Subjects

The methodology of the *Genetic and Environmental Risk Factors of Hemorrhagic Stroke *study have been previously reported [5,11]. Cases of potential ICH or SAH in the Greater Cincinnati and Northern Kentucky are identified by surveillance of 16 hospital emergency and radiology departments and through monitoring of hospital discharge diagnoses. Eligible cases are ≥ 18 years, are without trauma or brain tumor as the cause of hemorrhage, and reside within a 50-mile radius of the University of Cincinnati. A subset of cases was invited to enroll in a direct interview and genetic sampling arm of the study. The response rate was reasonable with over 60% of the cases agreeing to participate. Two controls for each interviewed case, matched by age (± 5 years), race, and gender were recruited from the general population through random digit dialing. Controls were informed of their participation in a risk factor study. Institutional Review Boards at each hospital approved the study, and informed consents were obtained from the participants.

SAH was defined as non-traumatic abrupt onset of severe headache or altered level of consciousness associated with blood in the subarachnoid space on CT or at autopsy, or with a clinical history and examination consistent with SAH where xanthochromia and increased red blood cells are found in the cerebrospinal fluid.

A total of 309 Caucasians were included for analysis, of which 107 were SAH cases matched to 202 controls. There was no significant difference in the average age of cases and controls (51.48 ± 12.93 yrs vs. 50.68 ± 12.23 yrs; *p *= 0.521) or gender distribution (64.4% vs. 64.8% female; *p *= 0.992) between the cases and the controls. We also genotyped samples from a small group of African American subjects. However, the limited sample size (24 cases and 43 matched controls) lacked sufficient power to identify associations. Thus, our results refer only to Caucasian cases and controls.

### DNA analysis

Buccal swabs were collected from each participant at the time of interview, and DNA was extracted by standard methods. Genotyping for *APOE *ε2/ε3/ε4 alleles was performed by RFLP [17]. For analysis of the SNP markers, genomic DNA was preamplified by whole genome amplification (WGA) using improved-primer extension preamplification. The WGA kit (High-Fidelity Expand Template System) was obtained from Roche Pharmaceuticals. Five nanograms of DNA was subjected to WGA and then diluted 50-fold, of which 2 μl was used for SNP genotyping. The WGA protocols are validated for analysis of genetic markers in our laboratory [18].

The TaqMan™ (fluorogenic 5' nuclease) assay was used for SNP genotyping. The primers and probes were obtained from Applied Biosystems. PCR was conducted in ABI 9700 thermocyclers, and the end-point results scored using the ABI 7900HT Sequence Detection System. In each 384-well plate, two reference samples and two negative controls were included for quality control.

We analyzed 12 SNPs spanning a 16 kb fragment on the *APOE *gene (Table 1) of which five upstream markers are now assigned locations on the *TOMM40 *gene [19]. Physical distance between the most distal 3'SNP (*rs10119*) on *TOMM40 *and the most proximal 5'SNP (*rs769446*) on *APOE *is approximately 2 kb. These 12 SNPs were analyzed in our previous study on ICH, which showed haplotypic association with lobar ICH [11]. We selected 10 SNPs on the *ELN *gene (Table 2), which were reported in previous association studies involving aneurysmal SAH [14-16].

**Table 1:** Distribution of the studied SNPs in the *APOE *gene region

**SNP No.**	dbSNP ID (*)	**NCBI Location**	**Genomic Location**
SNP1	*rs157581 *(TC)	17663932	*TOMM40 *EX2
SNP2	*rs1160983 *(GA)	17665447	*TOMM40 *EX5
SNP3	*rs1160985 *(TC)	17671630	*TOMM40 *IN5
SNP4	*rs1160984 *(CT)	17672142	*TOMM40 *IN5
SNP5	*rs10119 *(GA)	17674891	*TOMM40 *3'UTR
SNP6	*rs769446 *(AT)	17676846	*APOE *Promoter
SNP7	*rs405509 *(TC)	17677054	*APOE *Promoter
SNP8	*rs440446 *(CG)	17677385	*APOE *5'UTR
SNP9	*rs769452 *(CT)	17679328	*APOE *EX2
SNP10	*rs429358*(TC)	17680159	*APOE *EX4
SNP11	*rs769455 *(TC)	17680258	*APOE *EX4
SNP12	*rs7412 *(CT)	17680297	*APOE *EX4

**Table 2:** Distribution of the studied SNPs in the *ELN *gene

**SNP No.**	dbSNP ID (*)	**NCBI Location**	**Genomic Location**
SNP1	*rs3757584 *(GT)	11474039	5' UTR
SNP2	*rs868005 *(AG)	11478458	IN1
SNP3	*rs2301995 *(CT)	11485484	IN4
SNP4	*rs2301994 *(CT)	11485607	IN4
SNP5	*rs3801459 *(AC)	11487753	IN4
SNP6	*rs13229379 *(AC)	11489504	IN5
SNP7	*rs2239691 *(GA)	11502513	IN19
SNP8	*rs2071307 *(CT)	11504058	EX20
SNP9	*rs104272300 *(GA)	11507178	IN22
SNP10	*rs3757587 *(CT)	11514372	IN31

(*) In Tables 1 and 2, the second nucleotide position under dbSNP ID represents the minor allele.

### Statistical methods

Allele frequencies were estimated by gene counting. Conformity of genotype proportions to Hardy-Weinberg equilibrium (HWE) was tested by a goodness-of-fit χ^2 ^test. Haplotype frequencies and probabilities of haplotype pairs for each individual were estimated by PHASE version 2.1. PHASE implements Bayesian methods for estimating haplotypes from population data [20]. Allele and genotype frequency differences were tested using allelic and genotypic χ^2 ^tests, respectively. Haplotype associations were tested using χ^2 ^and haplotype trend regression (HTR) [21]. All *p*-values for allele, genotype and haplotype associations were empirically determined by Monte Carlo simulations as described by Becker and colleagues [22,23]. Multiple testing was accounted for by testing a global hypothesis of no association for each of the single locus and haplotype test [22,23].

### Genomic control

We performed a genomic control analysis to adjust for population substructure [24]. This was done by typing 30 unlinked SNPs distributed throughout the genome as null markers in all samples (cases and controls) and conducting test statistics to estimate λ following the methods as described [25].

## Results

### Genetic variation at the *APOE *locus

Genotype and allele frequencies among the cases and the controls are presented in Table 3. Genotypes at all 12 markers were in HWE. We did not observe significant association either at allelic or genotypic level (global *p *= 0.452 and 0.807, respectively). Marginal frequency differences between cases and controls at allelic and genotype levels were observed in SNP 1 and 4 (Table 3). We performed a separate analysis for the isoformic ε2/ε3/ε4 alleles, which also revealed no significant association (Table 4).

**Table 3:** Distribution of genotypes and allele frequencies at the *APOE *gene in the Caucasian cases and controls

**Locus **	**Genotype**			**Freq(q)**	**Cases vs. Controls (*p*-value)**	
				
	**AA**	**Aa**	**aa**		**Allele**	**Genotype**
SNP1	70	32	1	0.165	0.056	0.142
	107	71	7	0.230		
SNP2	102	4	0	0.019	0.371	0.641
	184	11	1	0.033		
SNP3	23	53	25	0.490	0.518	0.738
	41	93	55	0.463		
SNP4	95	7	0	0.034	0.062	0.144
	162	22	3	0.075		
SNP5	55	41	5	0.252	0.570	0.589
	91	66	14	0.275		
SNP6	74	27	2	0.150	0.164	0.327
	124	69	4	0.195		
SNP7	81	19	0	0.095	0.536	0.503
	145	35	3	0.112		
SNP8	45	50	10	0.333	0.304	0.548
	75	95	26	0.375		
SNP9	104	1	0	0.005	0.289	0.760
	185	3	1	0.013		
SNP10	71	21	2	0.133	0.361	0.675
	130	48	6	0.163		
SNP11	105	0	0	-	0.145	0.145
	194	5	0	0.013		
SNP12	83	11	0	0.059	0.108	0.213
	149	34	1	0.098		
Global					0.452	0.807

Note: In Tables 3 and 6, Genotype 'aa' represents the minor homozygous genotype; the first row corresponding to each SNP marker presents the data on cases and the second row presents the data on controls.

**Table 4:** Distribution of APOE ε2/ε3/ε4 alleles and genotypes

	**Case (freq)**	**Control (freq)**	***P***
**Genotype**			
ε2/ε2	0	1 (0.005)	0.998
ε2/ε3	10 (0.106)	32 (0.174)	0.162
ε2/ε4	1 (0.011)	2 (0.011)	0.997
ε3/ε3	61 (0.649)	97 (0.527)	0.069
ε3/ε4	20 (0.213)	46 (0.250)	0.557
ε4/ε4	2 (0.021)	6 (0.033)	0.727
**Allele**			
ε2	11 (0.058)	36 (0.098)	0.143
ε3	152 (0.808)	272 (0.740)	0.073
ε4	25 (0.132)	52 (0.163)	0.386

We conducted an analysis to test for association at the haplotype level based on data from all 12 SNPs (Table 5). Using PHASE, we observed a total of 66 haplotypes. In Table 5, we present data on 9 common haplotypes (frequency >0.02 in either cases or controls as inferred by PHASE. TGTCGATCCTTC (Hap1) was inferred as the most common haplotype, which accounted for 30% and 19% of all haplotypes in cases and controls, respectively. Global tests of association were significant for both χ^2 ^test and HTR (*p *= 0.019 and 0.036, respectively). Among all haplotypes, Hap1 showed significant difference between cases and controls by both methods (Table 5).

**Table 5:** *APOE *haplotype frequencies inferred by PHASE and HTR and the corresponding p-values showing the levels of difference between cases and controls.

**Haplotype**	**Frequency**		***p***-_**CS**_	***p*-**_**HTR**_
	**Cases**	**Controls**		
TGTCGATCCTTC (Hap1)	0.296	0.194	0.001	0.001
TGCCGATGCTTC (Hap2)	0.148	0.140	0.689	0.123
CGCCAATCCTTC (Hap3)	0.074	0.068	0.704	0.216
TGCCATTGCTTC (Hap4)	0.047	0.053	0.821	0.793
TGTCGATCCCTC (Hap5)	0.050	0.040	0.438	0.203
TGCCGACGCTTC (Hap6)	0.029	0.024	0.958	0.168
TGCCATTCCTTC (Hap7)	0.035	0.025	0.521	0.086
TGTTGATCCTTC (Hap8)	0.030	0.048	0.264	0.813
TGCCGATGCCTC (Hap9)	0.021	0.034	0.309	0.647
Rare Haplotypes (<0.2)	0.271	0.374		
Global Test			0.019	0.036

***p***-_**CS **_: empirical p-values based on chi square test statistics, ***p***-_**HTR**_: empirical p-values based on haplotype trend regression test statistics.

**Figure 1:**
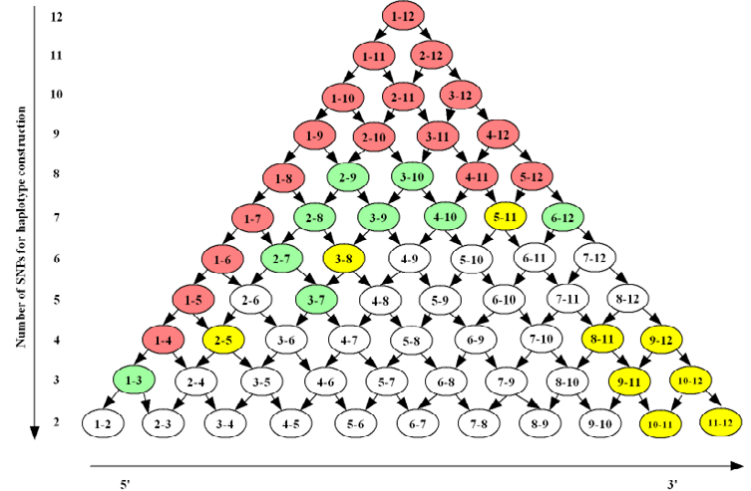
**Sliding Window Analysis of Hap1 (TGTCGATCCTTC)**. Each circle depicts the most common haplotype created by the SNPs indicated (e.g., circle 1–12 represents the most common haplotype formed by SNPs 1 to 12). Significance level of the most common haplotype is indicated by color: yellow (*p *~ 0.05–0.1), green (*p *~ 0.01–0.05) and red (*p *< 0.01). Global significance for sliding window is 0.013.

To explore the haplotype association at a finer level, we conducted a 'sliding haplotype' analysis to decompose Hap1, the most common and significantly associated haplotype. None of the other haplotypes showed significant association even with the sliding window analyses (data not shown), and they were consequently dropped from further analysis. A graphical representation of this analysis is presented in Figure 1. The uppermost circle in the figure, with the number '1–12', represents Hap1 inferred using all SNPs (SNPs 1 to12). Progressively smaller windows of this haplotype were formed at each level of the pyramid. These depict windows created with combinations of SNPs used in haplotype construction for that level (e.g., circle 1–11 represents the most common haplotype formed by SNPs 1 to 11, circle 2–12 represents the most common haplotype formed by SNPs 2 to 12, and so on). Haplotypes with significant associations are shown in yellow (*p *~ 0.05–0.1), green (*p *~ 0.01–0.05) and red (*p *< 0.01). A trend of ascending significance is observed at the 5' region. Marginal associations are also observed at the 3' region, where SNPs encoding the isoformic ε2/ε3/ε4 variants are located (SNP10 and SNP12 together form the ε2/ε3/ε4 variants – cytosine at both sites result in the ε4 isoform, thymine at the first site and cytosine at the second site form ε3, and thymine at both sites yield ε2).

### Genetic variation at the *ELN *locus

Genotype and allele frequencies of *ELN *SNPs among the cases with aneurysmal SAH and controls are presented in Table 6. Genotypes were in HWE with the exception of SNP2 and SNP7. One marker *rs13229379 *(SNP6) was found to be monomorphic in our population and was thus dropped from subsequent analysis. None of the SNPs were associated with SAH.

**Table 6:** Distribution of genotypes and allele frequencies at the *ELN *gene in the Caucasian cases and controls

**Locus**	**Genotype**			**Freq(q)**	**Cases vs. Controls (*p*-value)**	
				
	**AA**	**Aa**	**Aa**		**Allele**	**Genotype**
SNP1	79	11	0	0.061	0.851	0.774
	141	16	1	0.057		
SNP2	33	42	14	0.393	0.863	0.968
	56	71	26	0.402		
SNP3	79	11	0	0.061	0.729	0.906
	138	20	1	0.069		
SNP4	81	9	2	0.070	0.544	0.851
	144	22	4	0.088		
SNP5	76	11	0	0.063	0.860	0.574
	142	18	2	0.067		
SNP7	34	42	15	0.395	0.948	0.998
	63	76	27	0.391		
SNP8	35	43	14	0.386	1	0.875
	63	84	23	0.382		
SNP9	47	12	5	0.172	0.399	0.697
	83	24	14	0.215		
SNP10	77	12	3	0.098	0.297	0.027
	126	42	1	0.130		
Global Test					0.847	0.153

Using PHASE, we inferred haplotypes based on all 9 SNPs and did not observe significant associations (global *p *= 0.229 and 0.198; Table 7). There was no association of haplotypes even after excluding the two markers (SNP2 and SNP7) that were not in HWE (data not shown). Further, the sliding window analysis showed no association between *ELN *and SAH.

**Table 7:** *ELN *haplotype frequencies inferred by PHASE and HTR and the corresponding p-values showing the levels of difference between cases and controls.

**Haplotype**	**Frequency**		***p*-_**CS**_**	***p*-_**HTR**_**
	**Cases**	**Controls**		
GACCAGCGC(Hap1)	0.396	0.353	0.306	0.019
GGCCAATGC(Hap2)	0.295	0.261	0.480	0.029
GACCAGCAC(Hap3)	0.112	0.127	0.734	0.284
GGCCAATGT(Hap4)	0.063	0.068	0.936	0.232
TATTCGCAC(Hap5)	0.036	0.024	0.351	0.045
Rare Haplotypes	0.097	0.167		
Global Test			0.229	0.198

## Discussion

Although we did not observe significant association of the *APOE *variants and also could not confirm the association of *APOE *ε4 with SAH, we did find an association between SAH and the most common *APOE *haplotype, which occurred in nearly 1/3 of the SAH cases compared to 1/5 of controls. This haplotype included regulatory regions of the gene in the 5' untranslated region. Since variations of the 5' regulatory region are traditionally associated with decreased or increased expression of the gene, we hypothesize that regulation of *APOE *is the primary mechanism of association of this gene with SAH. We have not examined the 3' untranslated region and our most distal 3' SNP was within the last exon of the gene. Variations in the 3' untranslated region are associated with post-transcriptional processing and we are unable to comment upon variations in these regions.

Our recent study in lobar ICH demonstrated that in addition to the association of *APOE *ε4 with lobar ICH, *APOE *haplotypes, which include non-coding variants in regulatory regions, mediate the risk of lobar ICH [11]. These findings underscore the importance of regulatory variants, in addition to coding sequences, in understanding the genetic basis of complex diseases.

Haplotypes have the advantage of providing information not only on the relationship of disease to a single polymorphism, but also on variants that are in linkage disequilibrium with the markers tested. The selection of large haplotypes may lead to "over-partitioning" in which haplotypes with very small frequencies may be spuriously associated with the phenotype in question. In our study, the most common haplotype was associated with the phenotype, making over-partitioning a less-likely confounder.

The primary function of APOE in lipid metabolism is to mediate the interaction of lipid particles with LDL and APOE receptors. The involvement of *APOE *polymorphisms in lipid metabolism, Alzheimer's disease, and a host of cardiovascular and cerebrovascular diseases imply pleiotropic effects of the gene [6-8]. Although outcome studies have implicated *APOE *ε4 as a risk allele for cognitive impairment following subacute phase of aneurysmal SAH [26] and a recent meta-analysis showed marginal association of ε4 carriers with SAH [27], the biological role of APOE in the etiology of SAH remains unclear. Based on epidemiologic studies showing association of lower cholesterol levels to hemorrhagic stroke including subarachnoid hemorrhage [28-30], it may be speculated that lipid metabolism involving APOE contributes to risk of SAH or its adverse outcomes.

A significant advantage of our study is the inclusion of polymorphisms other than those that code for *APOE *ε2 and *APOE *ε4. The overall haplotype spans a large region of the 5' untranslated region and the exons of the *APOE *gene, which allows for an examination of the regulatory regions. The sliding window analysis provides a compelling indication of association, which emerges from the 5' region of the gene. To our knowledge, no other study has examined any other SNPs of the *APOE *gene and risk of SAH.

The association of *ELN *with SAH has not been consistent. Using an affected sib-pair design, Onda *et al*. [14] first reported linkage of familial IA to chromosome 7q11 in Japanese families. The putative locus was later confirmed by linkage in a set of extended pedigrees from Utah [31]. However, two other linkage studies, from Japan and Finland, did not replicate these findings [32,33]. A putative candidate gene, *ELN*, which maps to 7q11, raised the expectation of its association with IAs.

Although we did not find significant association either at allelic or haplotype levels, we do not to rule out the possible association of *ELN *with SAH. Differences in allele frequencies and haplotype structures among populations could influence association results. The allele frequencies for many SNPs in our population were different from those reported in the Japanese population [14]. Further, an associated polymorphism could be in LD with the functional variant and not be the functional variant by itself. Variation in LD pattern across populations, therefore, would be important in assessing association.

Case-control association designs should be viewed with caution because spurious association could be introduced by unrecognized population substructure. To guard against such false associations, we used a genomic control approach in which null markers distributed throughout the genome are used to adjust the association test statistics [24]. The adjustment is carried out by estimating a variance inflation factor, λ, from the distribution of the test statistics at the null loci. In the absence of substructure, λ is 1 and the genomic control approach is equivalent to a standard case-control test. A major strength of our study was that we used genomic control to evaluate and correct for any population substructure in our samples. The λ value obtained form the 30 null SNPs was 1.06. This clearly illustrates absence of any major population stratification in our samples, which suggests that our results of association or lack thereof remain valid.

## Conclusion

In conclusion, our study suggests a plausible role of the upstream regulatory region of *APOE *in the etiology of aneurysmal SAH. The complexity of the biological mechanisms underlying *ELN *in conjunction with genetic heterogeneity among ethnically diverse populations could influence our observed lack of association. Further studies with larger sample sizes and in additional ethnic groups are required to establish the likely involvement of *ELN *variants in SAH.

## Authors' contributions

RK, PP and HX carried out the molecular genetic analysis, data analysis and participated in the preparation of the manuscript. MH, LS were involved in the study conception, data management, quality control and recruitment of subjects. CM was the principal data manager, and involved in conceiving the variable definitions, data cleanup and baseline statistical analysis. PS was the principal statistical analyst for the non-genetic variables. BK, DK and MF were involved in conceptual study design, critique of the analyses and manuscript preparation. DW, JB, RC and RD were involved in conceiving and designing the study, preparation and final revision of the manuscript. All authors read and gave approval of the version to be published.

## Pre-publication history

The pre-publication history for this paper can be accessed here:


